# COVID-19 Presenting With Intractable Hiccups: A Literature Review and a New Case

**DOI:** 10.7759/cureus.78701

**Published:** 2025-02-07

**Authors:** Mohammed Aloqaily, Alaa Tarazi, Abdullah Ammar, Ibrahim Alfarrajin, Raed Ababneh, Wafi A Aloqaily, Yousef Alasaad

**Affiliations:** 1 Internal Medicine, University of Maryland Medical Center, Baltimore, USA; 2 Medicine, The University of Jordan, Amman, JOR; 3 Internal Medicine, Ascension Saint Agnes Hospital, Baltimore, USA; 4 Internal Medicine, Hamad Medical Corporation, Doha, QAT

**Keywords:** covid-19, critical care, hiccups, persistent hiccups, pulmonology, review

## Abstract

Hiccups manifest as involuntary and repetitive diaphragm contractions, often involving the intercostal muscles. However, the precise underlying mechanism remains incompletely understood but typically benign. During the COVID-19 pandemic, the predominant clinical presentation featured fever, cough, and dyspnea. However, multiple atypical presentations are increasingly recognized as manifestations of COVID-19, including refractory hiccups. This article aims to examine the shared characteristics, distinctions, notable correlations, and prognosis among COVID-19 patients presenting with intractable hiccups. Additionally, we present a 79-year-old male with a history of Parkinson’s disease, hypertension, and diabetes who presented with refractory hiccups, cough, and a runny nose. Laboratory analysis revealed elevated inflammatory markers and a positive COVID-19 test. The patient responded well to medical management, and the hiccups were resolved.

Notably, the association between persistent hiccups and COVID-19 infection is increasingly recognized, predominantly affecting older males with comorbidities and can be the sole complaint. Furthermore, we analyzed 29 cases of COVID-19 with persistent hiccups in the English literature. The mean duration of symptoms was 3.9 days with the majority of these cases being males (96.55%) and an average age of affected individuals of 58.28 years. Cough was the most frequently associated symptom (31.03%), while an equal proportion of patients (31.03%) reported intractable hiccups as their sole complaint. Additionally, common findings included elevated inflammatory markers, electrolyte imbalances, and infiltrates on imaging. Most of the patients demonstrated substantial improvement through symptomatic and medical management; however, mortality was documented in two cases, which highlights the potential for this seemingly benign manifestation to mislead and necessitate thorough evaluation upon presentation.

## Introduction

Hiccups, scientifically referred to as hiccoughs or singultus, manifest as involuntary and repetitive contractions of the diaphragm, often involving the intercostal muscles. However, the precise underlying mechanism remains incompletely understood [1]. Typically, hiccups are transient and benign episodes that resolve spontaneously [2]. They can be categorized based on duration, with acute hiccups lasting less than 48 hours, persistent hiccups persisting for over two days, and intractable hiccups enduring for more than a month [2]. Persistent hiccups may signal serious underlying conditions, such as central nervous system or gastrointestinal (GI) disorders [2,3]. Notably, in recent times, individuals diagnosed with COVID-19 have been observed to experience persistent hiccups, although this manifestation is rare [4].

Since the identification of an unusual pneumonia presentation linked to the novel coronavirus SARS-CoV-2 in December 2019 [5], the global community has grappled with the COVID-19 pandemic, significantly impacting various facets of individuals' lives. Some patients exhibit GI symptoms and nonspecific respiratory manifestations, including delirium, fatigue, and loss of appetite, underscoring the diverse clinical presentation of COVID-19 [6].

During the COVID-19 pandemic, the predominant clinical manifestations were fever, cough, and dyspnea [7]. However, the evolving nature of the disease revealed a diverse range of manifestations affecting various physiological systems, including cardiovascular, GI, and neurological systems. Some patients exhibited GI symptoms and nonspecific manifestations, such as delirium, fatigue, and loss of appetite, further highlighting the varied clinical presentations of COVID-19 [6]. Remarkably rare manifestations were also documented, such as persistent or refractory hiccups [7,8].

In current medical literature, intractable hiccups are increasingly recognized as a notable manifestation of COVID-19, with approximately 29 documented cases upon our review. While generally benign, these hiccups can serve as the exclusive indicator of underlying serious pathology, as evidenced by being the sole presentation in reported cases that, unfortunately, ended with patient demise [7]. This underscores the imperative for heightened attention and seriousness when confronted with persistent and intractable hiccups.

Motivated by these considerations, our article embarked on elucidating the shared characteristics, distinctions, notable correlations, and prognosis among COVID-19 patients presenting with intractable hiccups, drawing attention to the significance of such cases. Furthermore, we present the inaugural documented case encountered in Qatar, adding a novel dimension to the evolving understanding of COVID-19 manifestations.

## Case presentation

A 79-year-old male with a medical history of well-controlled Parkinson's disease (on carbidopa-levodopa), controlled hypertension, diabetes mellitus, and dependent on a nasogastric tube (NGT) for the past year prior to his admission, who was up to date with COVID-19 vaccination, presents to the emergency department (ED) with complaints of cough, runny nose, and persistent hiccups.

The patient reported experiencing relentless hiccups for several weeks, subsequently developing a cough with whitish sputum production and a runny nose. Initially, he did not seek help as he self-managed the symptoms with a brief course of amoxicillin- clavulanic acid (875/125 mg twice daily), and a cough suppressant (dextromethorphan), noting improvement. However, he presented to the ED as the hiccups persisted accompanied by a recurrence of cough, runny nose, and fever, documented as 37.5°C. Notably, upon presentation to the ED, he was febrile, with a high axillary temperature measuring 39.6°C. His other vital signs were stable (heart rate: 80; respiratory rate: 18; blood pressure: 119/59; and O_2_ sat: 96% on room air), and physical examination was unremarkable except for the presence of NGT, and his chest examination revealed good bilateral air entry without added sounds.

**Figure 1:**
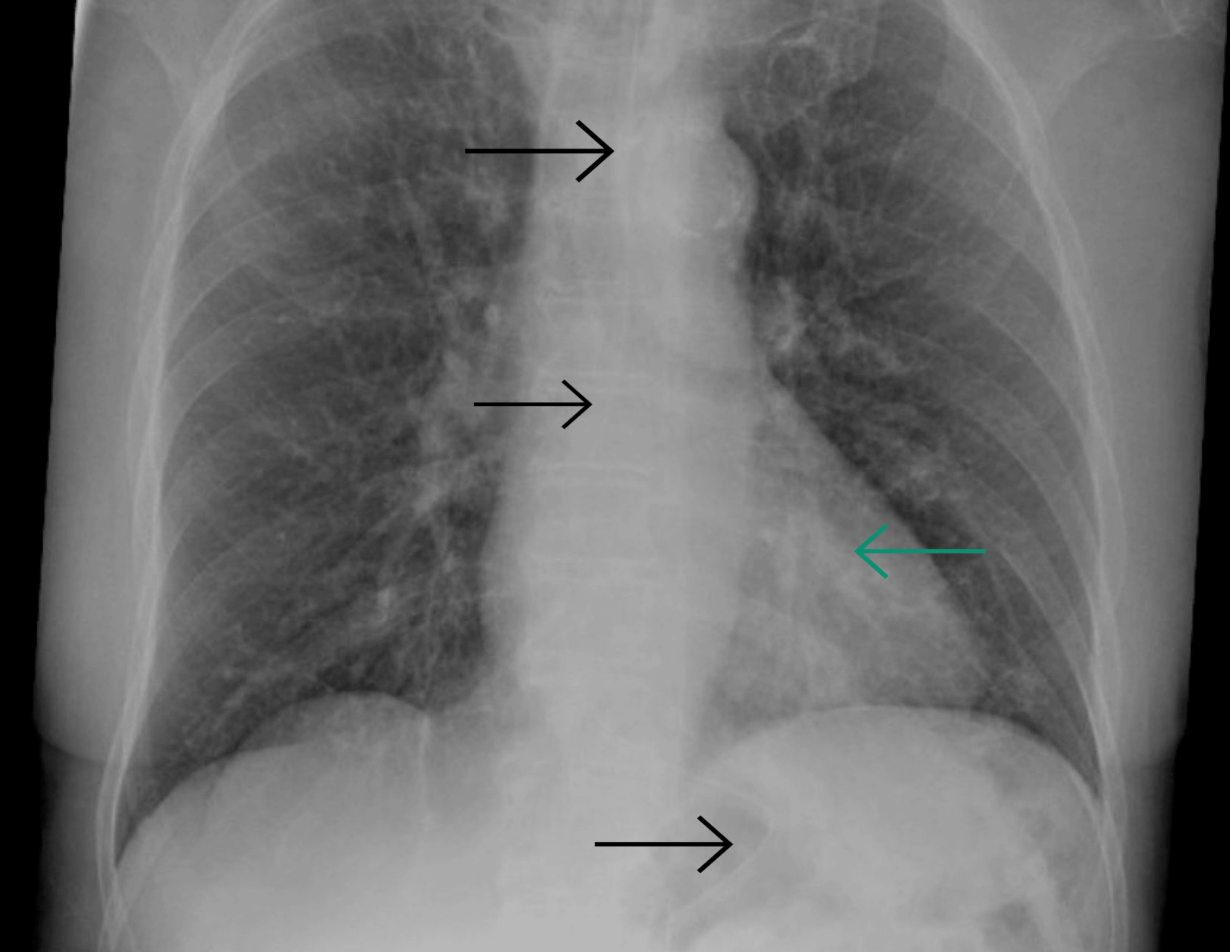
Chest X-ray showing suspected infiltrates in the left lower lung zone, in the retrocardiac location, with clear costophrenic angles. Black arrows indicate the nasogastric tube.

Further investigation through laboratory analysis revealed a white blood cell (WBC) count of 6.6, an elevated C-reactive protein (CRP) level of 33, a urea level of 10, negative blood cultures, and positive results on COVID-19 antigen and PCR tests (Table 1). Noteworthy in this context, a chest X-ray (CXR) exhibited suspected infiltrates in the left lower lung zone (Figure 1). Consequently, the patient was admitted and received medical management for COVID-19 pneumonia (remdesivir and dexamethasone) and baclofen for his hiccups, leading to symptoms amelioration and uncomplicated discharge to home. 

**Table 1:** Relevant laboratory findings and reference ranges. CRP, C-reactive protein; WBC, white blood count; AST, serum aspartate aminotransferase; ALT, alanine aminotransferase

Lab	Patient values at presentation	Reference range
WBC	6.6x10^3^/uL	4-10
Hemoglobin	9.6 gm/dL (as baseline)	13-17
Platelets	172x10^3^/uL	150-410
Urea	10.0 mmol/L	2.5-7.8
Creatinine	51 umol/L	62-106
Sodium	140 mmol/L	133-146
Potassium	3.9 meq/L	3.5-5.3
CRP	33.0 mg/L	0-5
Adjusted calcium	2.4 mmol/L	2.2-2.6
AST	20 uint/L	8-48
ALT	16 unit/L	0-56
Bilirubin	6 mmol/L	5.1-17
Albumin	33 gram\L	35-55

Despite knowing that persistent hiccups can be induced by multiple conditions and may rarely present as a non-motor symptom of Parkinson's disease, what supports our case are the facts that he had Parkinson's disease for many years, it was well-controlled with carbidopa-levodopa as mentioned, and he did not exhibit other signs of Parkinsonism, as his disease was controlled. More importantly, the hiccups were new for the patient, starting after the onset of other upper respiratory tract signs and symptoms, and resolving after he received COVID-19 treatment (remdesivir-dexamethasone), although no changes were made to the management of Parkinson's disease. Additionally, it is unlikely that the hiccups were caused by the NGT, as the patient had been dependent on it for a year before the onset of his symptoms, it was not changed, and he was discharged without any other intervention, but with symptom improvement. The temporal relationship of the symptoms to the upper respiratory tract (URT) symptoms, the positive COVID-19 test, and the resolution of symptoms following COVID-19 treatment, while maintaining the same management for other conditions, supports the causality in our case.

## Discussion

SARS-CoV-2 is a newly encountered microorganism that resulted in a global pandemic causing high morbidity and mortality among populations [7,8]. Patients who were infected presented with various symptoms, with fever, cough, and shortness of breath being the most frequently encountered [9]. Additionally, patients can present with non-respiratory symptoms such as abdominal pain and diarrhea. Notably, other rare presentations affecting various systems have been reported and continue to emerge, including myocardial infarction, meningoencephalitis, and, surprisingly, intractable hiccups [9,10].

This review delves into the correlation between persistent hiccups and COVID-19 infection, synthesizing data from 22 reports involving 29 patients. Hiccups present as audible spasms of the diaphragm and intercostal muscles, frequently accompanied by sudden glottic contractions [3]. Pathophysiologically, hiccups involve one of the three components of the hiccup reflex arc: afferent fibers, predominantly comprising the peripheral phrenic, vagus, and sympathetic nerves (T6-T12), which are responsible for transmitting sensory signals from somatic and visceral organs to the central unit in the brainstem [11]. Subsequently, the efferent pathway activates the motor fibers of the phrenic nerve, which govern the diaphragm, along with accessory nerves that innervate the intercostal muscles [11].

Despite continuous investigation, the precise mechanism underlying hiccups remains elusive, and the etiology is poorly understood, lacking specific triggers for acute episodes or definitive causes for persistent and intractable occurrences [1]. Acute hiccups may be provoked by factors such as consuming a large meal, spicy foods, tobacco smoke, and other irritants affecting the GI or pulmonary systems, alongside psychosomatic factors like stress and anxiety [1]. Persistent hiccups may stem from diverse origins, including pharmacological factors, with relatively frequent incidences associated with central nervous system disorders such as brain tumors or intracranial injuries, often accompanied by neurological symptoms [12].

Pneumonia has historically been considered an infrequent cause of hiccups, with documented cases tracing back to 1951 when Dr. Laha et al. in India reported the initial instance of pneumonia manifesting as a "hiccough" [13,14]. However, in recent times, particularly amid the COVID-19 pandemic, there has been a noticeable increase in such occurrences. The first recorded case of persistent hiccups linked to COVID-19 was documented in July 2020 by Prince and Sergel, involving a 62-year-old male with COVID-19 pneumonia [4]. Despite the uncertain connection between hiccups and COVID-19, other bacterial and viral ailments such as Helicobacter pylori, influenza, herpes zoster, neurosyphilis, and tuberculosis have been associated with inducing hiccups [2].

Our search and analysis of the English literature unveiled 29 COVID-19-related cases characterized by persistent hiccups with a mean duration of 3.9 days, predominantly concentrated in Asian nations (58.62%). Notably, the majority of these cases were male (96.55%), with only one patient being female. The average age of affected individuals was 58.28 years, spanning a range from 22 to 87 years, and 13.8% were identified as either current or former smokers. Hypertension emerged as the most prevalent comorbidity (44.83%), followed by a history of cardiovascular events (20.69%) (Table 2 and Table 3). 

**Table 2:** Characteristics and presentations of patients with COVID-19 who presented with refractory hiccups upon reviewing the literature. NA, not applicable; HTN, hypertension; DM2, diabetes mellitus type 2; IHD, ischemic heart disease; CABG, coronary artery bypass grafting; CRP, C-reactive protein; GFR, glomerular filtration rate; Hg, hemoglobin; Hct, hematocrit; PLT, platelets; SPO2, oxygen saturation; LDH, lactate dehydrogenase; Na, sodium; Hb, hemoglobin; CT, computed tomography; CXR, chest x-ray; ECHO, echocardiogram; LV, left ventricle; BUN, blood urea nitrogen; BNP, B-type natriuretic peptide; PD, peritoneal dialysis; ESKD, end-stage kidney disease; SOB, shortness of breath; WBCs, white blood cells; ALT, alanine aminotransferase; AST, aspartate aminotransferase; ALP, alkaline phosphatase; FBS, fasting blood sugar; RBCs, red blood cells; CSF, cerebrospinal fluid; GABA, gamma-aminobutyric acid; OSA, obstructive sleep apnea; CTPA, computed tomography pulmonary angiography

Source	Age (gender), smoking status	Comorbidities	Laboratory findings	Covid-19 symptoms	Hiccups duration	Outcome	Therapy	Imaging findings
Prince, Sergel, 2020 (USA) [4]	62 (M), non-smoker	Diabetes, HTN, coronary artery disease	Leukopenia, thrombocytopenia, hyponatremia, and hypochloremia	Fever, tachycardia, and weight loss	4 days	Symptoms improved	Ceftriaxone, azithromycin, and hydroxychloroquine	CXR reveals a ground glass opacity in the right and left lung. CT scan demonstrating scattered ground glass opacities in the upper and lower lung lobes
Habadi et al., 2021 (Saudi Arabia) [7]	64 (M), ex-smoker	DM2, HTN, IHD, post-CABG in 2017, dyslipidemia, peripheral vascular disease, erectile dysfunction	↑ CRP, ↓ GFR, uremia, ↓ Hg, ↓ Hct, and thrombocytopenia	Runny nose, cough, fatigue, and dizziness	5 days	No improvement, the patient died	Azithromycin, cefuroxime, dexamethasone, enoxaparin sodium, and levofloxacin	CXR revealed bilateral infiltrates, which later progressed to right chest wall surgical emphysema and rim pneumothorax. ECHO showed limited echo views and mild concentric left ventricular hypertrophy
Bakheet et al., 2020 (Egypt) [8]	48 (M), non-smoker	HTN	Tachypnea, ↑ CRP, ↑ ferritin, and ↑ LDH	Sore throat and fever	7 days	Improved symptoms	Ceftriaxone, azithromycin, hydroxychloroquine, oseltamivir, ascorbic acid, zinc, antipyretics, prophylactic anticoagulation, PPI, domperidone, and baclofen	CT of the chest showed bilateral subpleural areas of ground-glass attenuation and a crazy paving pattern. The abdominal US was unremarkable, apart from gaseous colonic distention.
Alvarez-Cisneros, Lara-Reyes, and Sanson-Tinoco, 2021 (Mexico) [10]	48 (M), NA	NA	Hyperglycemia, thrombocytopenia, lymphopenia, leucopenia, and SPO_2_ = 93% (hypoxemia)	NA	4 days	No improvement	Metoclopramide, omeprazole, ondansetron, and frappemagaldrate/dimeticone	CXR revealed bilateral infiltrates. Thoracic CT scan showed multiple zones of diffuse alveolar infiltrates across all segments of both lungs
Talwar et al., 2021 (India) [15]	49 (M), non-smoker	HTN	↑ D-dimer, ↑ RR, ↑ urea, ↓ Hb, ↓ Na, and ↓ PLT	Fever	3 days	Symptoms improved	Dexamethasone, ivermectin, low molecular weight heparin, and remdesivir	All cases showed ground-glass opacities involving the lower lobes, suggestive of COVID-19 infection
	22 (F), non-smoker	NA	↑ D-dimer, ↑ RR, ↑ urea, ↓ Hb, ↓ Na, and ↓ PLT	NA	5 days	Symptoms improved	Dexamethasone, ivermectin, low molecular weight heparin, metoclopramide, and remdesivir	
	70 (M), non-smoker	NA	↑ CRP, ↑ D-dimer, ↑ ferritin, ↑ PLT, ↑ total bilirubin, ↑ urea, and ↓ Hb	Weight loss	8 days	Symptoms improved	Antibiotics, baclofen, dexamethasone, ivermectin, low molecular weight heparin, and remdesivir	
Bacharaki et al., 2022 (Greece) [16]	70 (M), NA	ESKD, PD, carpal tunnel syndrome, ischemic heart failure	↑ CRP, ↓ Hb, ↑ troponin, and ↑ ferritin	Anorexia, nausea, vomiting tendency, weight loss, and NSTEMI	2 days	Improved symptoms	Dual antiplatelet therapy, enoxaparin, glucose-based PD exchanges, potassium supplementation, metoclopramide, chlorpropamide, and baclofen	Chest CT showed signs of mild pneumonia (lung infiltration in the right upper lobe, small areas of ground glass opacities, and small areas of atelectasis)
Jaishi et al., 2022 (Oman) [17]	72 (M), smoker	HTN	↑ D-dimer, ↑ ferritin, ↑ LDH, ↑ CRP, and ↑ lactic acid	Epigastric pain and fever	5 days	Improved symptoms	Pantoprazole, ceftriaxone, azithromycin, prednisolone, and metoclopramide	CXR revealed bilateral mid-lung opacities, while chest CT showed ground-glass opacity with mosaic attenuation, vascular dilation, and a few areas of air bronchogram in both lungs.
K. Pandey, Pandey, and Andrews, 2022 (USA) [18]	62 (M), NA	NA	Hyponatremia, hypokalemia, ↑ D-dimer, ↑ CRP, and ↑ WBCs	Headache, mild dyspnea, nausea, and vomiting	2 days	Symptoms improved	IV bolus of 3% saline solution followed by continuous infusion	NA
Xiang et al., 2021 (China) [19]	56 (M), NA	HTN	SPO_2_ 85%, CSF pressure > 330 mmH2O, and ↑ protein level in CSF analysis	Fever, fatigue, and dizziness	4 days	Symptoms improved	Oxygen, lopinavir/ritonavir, interferon alpha-2b, moxifloxacin, gamma globulin, mannitol dehydration, chlorpromazine, midazolam, and methylprednisolone	CT scan of the chest showed scattered and patchy ground-glass opacities in both lungs
Atiyat et al., 2021 (USA) [20]	61 (M), smoker	HTN	D-dimer, ↑ ferritin, ↑ LDH, ↑ CRP, ↑ lactic acid, and ↑ procalcitonin	Intermittent sharp mid-sternal chest pain and fever	2 days	Improved symptoms	Ceftriaxone, azithromycin, pantoprazole, dexamethasone, and metoclopramide	CXR displayed bilateral mid-lung opacities, more prominent on the right than the left, consistent with multifocal pneumonia
Sangamesh et al., 2021 (India) [21]	72 (M), NA	HTN, diabetes	↑ CRP and ferritin, ↑ LDH, ↑ urea, hyponatremia, and SPO_2_ 92%	NA	5 days	Symptoms improved	Antipyretics, favipiravir, baclofen, acebrophylline, levocetirizine, montelukast, N-acetylcysteine, oral antibiotics, oseltamivir, PPIs, short-acting insulin, steroids, vitamin D & C, and zinc	CXR shows bilateral lower lobe infiltrates
Albtoosh et at., 2023 (Jordan) [22]	82 (M), smoker	HTN, hyperlipidemia, CKD	↑ BNP, ↑ creatinine, SPO_2_ 89%, ↓ Hb, ↑ LDH, ↑ D-dimer, and ↑ CRP	Dry cough, orthopnea, fever, and hypotension	5 days	Sudden cardiac arrest with asystole	Antipyretics, IV fluids, noradrenaline infusion, IV antibiotics, oxygen therapy, rate control agents, and anticoagulation	CXR revealed bilateral lower zone infiltrates, with continued deterioration until the patient passed away
Karampoor, Afrashteh, and Laali, 2021 (Iran) [23]	58 (M), NA	NA	Thrombocytopenia, uremia, ↑ LDH, ↑ ferritin, tachypnea, SPO_2_ = 90% (hypoxemia), and acidosis	Pain, myalgia, fever, and dry cough	6 days	Improved symptoms	Remdesivir, dexamethasone, famotidine, zinc, vitamin C, diphenhydramine syrup, and prednisolone	CT showed a brief involvement of the lung in the form of ground-glass opacity
Bîrluțiu and Sofariu, 2022 (Romania) [24]	46 (M), non-smoker	NA	↑ CRP, mild elevation in ALT, ↑ ferritin, ↑ IL-6, and ↑ fibrinogen	Low-grade fever, headache, chills, abdominal pain, dry cough, insomnia, and lack of appetite	5 days	Symptoms improved	Dexamethasone, ivermectin, enoxaparin, pantoprazole, paracetamol, metoclopramide, remdesivir, ondansetron, and drotaverine hydrochloride	CT scan showed multiple areas of ground-glass opacity, disseminated bilaterally, both peripherally and in the upper lobes, with alveolar consolidation occupying 5% of the right upper lobe, 5% of the left upper lobe, and 25% of the left lower lobe
Ikitimur et al., 2021, (Turkey) [25]	60 (M), Non-smoker	NA	↑ ALT, ↑ CRP, ↑ ferritin, ↓ Hb, neutrophilia, tachypnea, uremia, and lymphocytopenia	NA	3 days	Improved symptoms	Avipiravir, dexamethasone, azithromycin, and chlorpromazine	CXR and CT revealed small ground-glass nodules scattered across both lungs, suggestive of viral pneumonia
	68 (M), Non-smoker	HTN, childhood poliomyelitis	↑ CRP, neutrophilia, tachypnea, uremia, ↓ Hb, and lymphocytopenia	NA	4 days	Improved symptoms	Ceftriaxone, chlorpromazine, enoxaparin, favipiravir, metoclopramide, and plaquenil	CT chest scan showing bilateral opacities
Ezeigwe et al., 2022, (UK) [26]	65 (M), NA	IHD, DM2, HTN, OSA, and central obesity	Hypoxia, tachycardia, ↑ CRP, ↑ lactate, and leukocytosis	Body aches and fever	5 days	Improved symptoms	Gaviscon, casirivimab, imdevimab, and dexamethasone	CXR showed widespread bilateral opacities (in the middle and lower zones), while CTPA demonstrated patchy ground-glass shadowing suggestive of pneumonitis and an incidental finding of a pulmonary hamartoma
Chiquete et al., 2021 (Mexico) [27]	62 (M)	HTN, hypercholesterolemia, hypertriglyceridemia, and mild vascular cognitive impairment.	↑ CRP, ↑ D-dimer, ↑ ferritin, ↑ LDH, and 92% SPO_2_ (hypoxemia)	Cough, mild dyspnea, and fever	5 days	Symptoms improved	Azithromycin, dexamethasone, enoxaparin, hydroxychloroquine, ivermectin, levomepromazine, and levosulpiride	CT scan showing COVID-19 pneumonia
Ali, Muturi, and Sharma, 2021 (Kenya) [28]	65 (M), non-smoker	Diabetes and HTN	Lymphopenia, ↑ CRP, and ↑ D-dimer	NA	7 days	Symptoms improved	Baclofen and GABA receptor agonist	High-resolution CT reveals peripheral ground-glass opacities
Sene et al, 2021 (Brazil) [29]	29 (M), non-smoker	NA	↑ CRP and lymphopenia	Cough, rhinorrhea, and mild SOB	2 days	Symptoms improved	Chlorpromazine	CT-scan showing peripheral ground opacities in lungs
Nakaya et al., 2021 (Japan) [30]	65 (M), non-smoker	Pneumonia	NA	Cough	3 days	Symptoms improved	Favipiravir and clonazepam	NA
	34 (M), non-smoker	Pneumonia	NA	Cough	1 day	Symptoms improved	Remdesivir	NA
	56 (M), non-smoker	Pneumonia	NA	NA	2 days	Symptoms improved	Remdesivir and chlorpromazine	NA
	87 (M), non-smoker	Pneumonia	NA	NA	3 days	Symptoms improved	Favipiravir and chlorpromazine	NA
	45 (M), non-smoker	Pneumonia	NA	Cough	2 days	Symptoms improved	Remdesivir and timepidium bromide	NA
Dorgalale et al., 2020 (Iran) [31]	52 (M)	Diabetes and congenital factor V deficiency	↑ ALP, ↑ ALT, ↑ AST, ↑ CRP, ↑ FBS, and ↑ RBCs	NA	>2 days	Symptoms improved	Chlorpromazine, metoclopramide, vitamin B12 and B complex, and vitamin C	CT-scan showed ground-glass opacities in the left lower lobe
Totomoch-Serra, Miramon, Manterola, 2021 (Chile) [32]	60 (M)	Hypercholesterolemia	↑ D-dimer, ↑ GGT, ↑ LDH, ↓ calcium, ↓ sodium, and 87% SPO_2_ (hypoxemia)	Dysgeusia and fever	>2 days	Symptoms improved	2% lidocaine, acetaminophen, clonazepam, haloperidol, ilaprazole, and metoclopramide	CXR showed lung parenchyma with decreased radiolucency and poorly defined, irregular edges, likely related to peribronchial thickening

**Table 3:** Descriptive analysis of patient characteristics in reported COVID-19 cases with refractory hiccups and their outcomes. NA, not applicable; HTN, hypertension; DM, diabetes mellitus; ESRD, end-stage renal disease; PD, peritoneal dialysis; OSA, obstructive sleep apnea

Variable	Number (%)
Gender	
Male	28 (96.55%)
Female	1 (3.45%)
Average age	58.28 years (22-87)
Smoking status	
NA	10 (34.48%)
Non-smoker	15 (51.72%)
Ex-smoker	1 (3.45%)
Smoker	3 (10.35%)
Hiccups average duration	3.9 days
Cases location by continent	
Asia*	17 (58.62%
North America	5 (17.24%)
Africa	2 (6.90%)
Europe	3 (10.34%)
South America	2 (6.90%)
*Turkey counted in Asia based on population majority	
Co-morbidities	
HTN	13 (44.83%)
Cardiovascular diseases	6 (20.69%)
DM	4 (13.79%)
Dyslipidemia	4 (13.79%)
ESRD	2 (6.90%)
Erectile dysfunction	1 (3.45%)
Childhood poliomyelitis	1 (3.45%)
PD	1 (3.45%)
Carpal tunnel	1 (3.45%)
OSA	1 (3.45%)
Central obesity	1 (3.45%)
Congenital factor V deficiency	1 (3.45%)
Vitals	
Fever	12 (41.38%)
Hypoxemia	8 (27.59%)
Tachypnea	6 (20.69%)
Tachycardia	2 (6.90%)
Hypotension	1 (3.45%)
Clinical outcome	
Improvement	26 ( 89.66%)
No improvement	1 (3.45%)
Death	2 (6.90%)

Examining the clinical presentation, cough was the most frequently associated symptom (31.03%), while an equal proportion of patients reported intractable hiccups as their sole complaint. Clinical evaluations revealed that a significant portion of patients exhibited fever (41.38%), hypoxia (27.59%), or tachypnea (20.69%), accompanied by elevated inflammatory markers. Additionally, thrombocytopenia and hyponatremia were each documented in 20.69% of cases (Table 2 and Table 4).

**Table 4:** Associated findings in patients presenting with COVID-19 and refractory hiccups. NSTEMI, non-ST-elevation myocardial infarction

Presentation	Number
N/A	9
Cough	9
Dyspnea	3
Weight loss	3
Nausea	3
Runny nose	2
Fatigue	2
Headache	2
Dizziness	2
Chest pain	1
Sore throat	1
Anorexia	1
Myalgia	1
Generalized pain	1
NSTEMI	1
Epigastric pain	1
Body aches	1
Vomiting	1
Chills	1
Abdominal pain	1
Insomnia	1
Orthopnea	1
Dysgeusia	1

It is noteworthy that nearly all patients who underwent imaging exhibited findings indicative of infection. In terms of prognosis, a majority of patients demonstrated substantial improvement through symptomatic and medical management. Unfortunately, mortality was reported in two cases (6.9%), emphasizing the imperative for vigilant observation and comprehensive treatment, as the clinical presentation might be deceiving (Table 2 and Table 3).

Lee et al. reported a higher prevalence of hiccups in males, particularly among those with non-CNS origins of persistent hiccups. They hypothesized that this increased susceptibility in males could be attributed to a lower synaptic threshold and heightened stimulation of the afferent or efferent nerves involved in the hiccup reflex pathway. Notably, even in the context of COVID-19, males demonstrate a greater propensity for developing hiccups [33]. This finding is substantiated by all the cases encompassed in this investigation, including the case of our patient. However, one instance involved a 22-year-old female who exhibited persistent hiccups associated with COVID-19, as documented by Talwar et al. [15]. It is noteworthy that hiccups can affect individuals across all age groups; in our analysis, there were 29 cases of COVID-19-related hiccups, exclusively observed in patients aged between 22 and 87 years, with an average age of 58. Our patient was 79 years old. Notably, patients who contracted COVID-19 and experienced refractory hiccups typically presented with hiccups prior to the diagnosis of the disease.

In addition, the majority of cases analyzed in our research were non-smokers (see Table 2), although one study suggests that smoking might contribute to the onset of hiccups, though the precise mechanism remains poorly understood [34]. Furthermore, a notable portion of the patients in our study had a history of hypertension, including our patient. It is also worth mentioning that another case report highlighted an inferior myocardial infarction in a 74-year-old male, where persistent hiccups were the primary presenting symptom, suggesting that hiccups could be induced by irritation of the phrenic nerves near the diaphragm [35].

Along with its respiratory manifestations, SARS-CoV-2 infection can present with various atypical extra-pulmonary symptoms, including GI symptoms such as diarrhea, nausea, vomiting, and abdominal pain. It has been reported that vaccinated individuals exhibit a higher frequency of these symptoms compared to unvaccinated individuals [36]. Supporting this, one study detailed the case of a 72-year-old man who initially presented with fever and epigastric burning pain after receiving two doses of Vero cell vaccination against SARS-CoV-2 [17]. Other GI symptoms, such as nausea and vomiting, were reported by Pandey et al. in a 62-year-old male, which was atypical for a COVID-19 presentation [18]. Moreover, human coronaviruses have the potential to invade the central nervous system through poorly understood mechanisms, resulting in various neurological symptoms like dizziness, headache, seizures, and altered mental state [37]. Xiang et al. described a case of a 56-year-old man who initially complained of fatigue and dizziness, later experiencing persistent hiccups, maxillofacial spasms, and increased muscle tension. These neurological symptoms were attributed to the detection of coronavirus-2 in the cerebrospinal fluid using metagenomic next-generation sequencing [19]. However, in our case, the patient presented with a cough and runny nose, typical COVID-19 symptoms, along with the notable addition of refractory hiccups.

A notable observation among the patients included in the study who experienced persistent hiccups is the presence of elevated inflammatory markers, such as CRP, along with the occurrence of acute kidney injury, as outlined in Table 1. Atiyat et al. documented elevated levels of all inflammatory markers, CRP, D-dimer, ferritin, and lactate dehydrogenase (LDH), in a COVID-19 patient who developed prolonged hiccups [20]. Similarly, Habadi et al. reported a 64-year-old male who initially presented with bilateral CXR infiltrates and elevated CRP levels, later experiencing a low glomerular filtration rate (GFR), indicating acute kidney injury [7]. Our patient also displayed elevated levels of urea and CRP in laboratory assessments.

Interestingly, additional investigations have uncovered cases of hyponatremia in patients presenting with persistent hiccups. Sangamesh et al. reported a 72-year-old male patient who experienced the uncommon co-occurrence of persistent hiccups and hyponatremia. They proposed that electrolyte imbalances could potentially lead to persistent hiccups, offering another plausible explanation for this atypical presentation [21]. Research by Jones et al. also indicated that hiccups can emerge as a symptom of hyponatremia [38]. Furthermore, the link between hyponatremia and COVID-19 was highlighted in one article [18].

It is also noteworthy that thrombocytopenia has been reported as a laboratory finding in the literature. The eHealth study, which focused on individuals experiencing hiccups alongside thrombocytopenia, analyzed FDA reports published on January 18, 2024 [39]. The study revealed that individuals over the age of 60 were most frequently affected by this condition. Among the medications commonly associated with thrombocytopenia and hiccups, according to eHealth data, ondansetron and omeprazole were administered in one case in the literature [39]. Additionally, dexamethasone, identified as a common medication in the eHealth study, was also given to two cases in the literature who developed thrombocytopenia, suggesting a potential association between these drugs and the condition.

The majority of reported cases showed symptom improvement after receiving appropriate medical treatment, including the case we presented, in which the hiccups were resolved. COVID-19 infection can lead to death through various pathways, including cardiac diseases [40] and respiratory infections [41]. Two COVID-19 cases with persistent hiccups ultimately developed cardiac arrest and subsequent fatality [7,22].

While physical therapy is considered the primary intervention for treating hiccups [23], the literature predominantly reports cases managed through medication-based approaches [9]. Notably, metoclopramide, chlorpromazine, and baclofen emerged as commonly used agents for addressing persistent hiccups (Table 1). Following treatment initiation, a significant proportion of patients showed improvement in symptoms (Table 1) [9]. Interestingly, in one documented case, medication proved ineffective, yet the hiccups resolved spontaneously alongside COVID-19 treatment, characterized by improved oxygenation and decreased inflammatory markers [20].

Pharmacological agents have also been implicated in triggering persistent hiccups, likely through their influence on the GI tract or central nervous system [1]. Although rare, certain medications, such as corticosteroids and benzodiazepines, are recognized as potential causes of this condition [42]. Notably, corticosteroids like dexamethasone have proven effective in reducing mortality among COVID-19 patients [43]. There have been instances where patients developed hiccup episodes following drug administration. For example, five patients undergoing chemotherapy developed hiccups five days after treatment initiation [44]. Another case involved a patient receiving dexamethasone for chemotherapy-induced nausea and vomiting (CINV), who subsequently experienced severe hiccups [45]. Among the reported cases, two patients developed hiccups during dexamethasone therapy for COVID-19; one patient's hiccups ceased after switching to prednisolone [23], while another required metoclopramide after transitioning to methylprednisolone for resolution [24]. It is worth noting that hiccups have been reported following treatment with azithromycin [7] and methylprednisolone [19], both identified as potential hiccup-inducing medications [46]. However, several cases in the literature involved patients receiving dexamethasone and other similar medications without developing hiccups, highlighting variability in individual responses [15,20,25-27]. These findings emphasize the importance of physicians remaining vigilant about potential side effects, including persistent hiccups, when prescribing medications such as corticosteroids to COVID-19 patients. This vigilance is crucial to ensuring patient comfort and achieving optimal treatment outcomes.

Finally, diagnostic imaging was reported in most cases, including CXR, computed tomography (CT), or a combination of both (Table 1). CXR and CT chest scans are widely recognized as rapid and accurate diagnostic tools for COVID-19, helping to monitor disease progression [47]. While most cases underwent both CXR and CT scans, CT scans were more frequently reported, likely due to their enhanced sensitivity and ability to provide detailed imaging with superior contrast [47,48]. Common radiographic findings included bilateral infiltrates on CXR and ground-glass opacities on CT scans. In contrast, our patient’s CXR revealed retro-cardiac opacity in the left lower lobe. This highlights the importance of thorough evaluation and imaging for such patients, as they may present with concerning findings despite a misleading presentation.

## Conclusions

Persistent hiccups have emerged as a notable, though uncommon, manifestation in COVID-19 patients, with a higher prevalence observed in males and older individuals, particularly those with underlying comorbidities such as hypertension (44.83%) and cardiovascular disease (20.69%). Our analysis of 29 reported cases reveals that these hiccups often occur alongside typical COVID-19 symptoms, such as cough (31.03%), or as the sole complaint (31.03%). Patients are also likely to present with fever (41.38%) and hypoxia (27.59%) upon evaluation. Additionally, radiographic imaging commonly showed bilateral infiltrates and ground-glass opacities, consistent with established patterns of COVID-19 pneumonia. Elevated inflammatory markers, such as CRP and urea, were frequent findings, and electrolyte imbalances, particularly hyponatremia, were observed in approximately 20% of cases.

While the majority of cases respond to symptomatic treatment and medical management, including the use of drugs like baclofen and metoclopramide, a small proportion of patients experienced severe outcomes, resulting in mortality (6.9%). In light of this, further studies are needed to establish a definitive link, and clinicians should remain vigilant for atypical COVID-19 presentations, including persistent hiccups, to ensure comprehensive patient evaluation and management, as they may signal more serious underlying conditions. Finally, an individualized approach is essential to ensure optimal patient outcomes, given the variability in responses.
